# Production of Minor Ginsenosides C-K and C-Y from Naturally Occurring Major Ginsenosides Using Crude β-Glucosidase Preparation from Submerged Culture of *Fomitella fraxinea*

**DOI:** 10.3390/molecules26164820

**Published:** 2021-08-09

**Authors:** Dae-Woon Kim, Won-Jae Lee, Yoseph Asmelash Gebru, Jitendra Upadhyaya, Sung-Ryong Ko, Young-Hoi Kim, Myung-Kon Kim

**Affiliations:** 1 Department of Food Science and Technology, Jeonbuk National University, Jeonju 54896, Korea; eodns3344@gmail.com (D.-W.K.); eugenelwjai@jbnu.ac.kr (W.-J.L.); yagebru@gmail.com (Y.A.G.); Jitu.upadhyaya@gmail.com (J.U.); Yhoi1307@hanmail.net (Y.-H.K.); 2 Institute for Liver and Digestive Diseases, Hallym University, Daehak-ro 1, Chuncheon 24252, Korea; 3 Department of Bioresource Engineering, McGill University, Montreal, QC 3A 0G4, Canada; 4 Bonghwang Cheonjong Ginseng Research Institute, Daegu 34544, Korea; Srko00316@naver.com

**Keywords:** mushroom, *Fomitella fraxinea*, enzyme preparation, major ginsenosides, biotransformation, compound-K, compound-Y

## Abstract

Minor ginsenosides, such as compounds (C)-K and C-Y, possess relatively better bioactivity than those of naturally occurring major ginsenosides. Therefore, this study focused on the biotransformation of major ginsenosides into minor ginsenosides using crude β-glucosidase preparation isolated from submerged liquid culture of *Fomitella fraxinea* (FFEP). FFEP was prepared by ammonium sulfate (30–80%) precipitation from submerged culture of *F. fraxinea*. FFEP was used to prepare minor ginsenosides from protopanaxadiol (PPD)-type ginsenoside (PPDG-F) or total ginsenoside fraction (TG-F). In addition, biotransformation of major ginsenosides into minor ginsenosides as affected by reaction time and pH were investigated by TLC and HPLC analyses, and the metabolites were also identified by UPLC/negative-ESI-Q-TOF-MS analysis. FFEP biotransformed ginsenosides Rb1 and Rc into C-K via the following pathways: Rd → F2 → C-K for Rb1 and both Rd → F2→ C-K and C-Mc1 → C-Mc → C-K for Rc, respectively, while C-Y is formed from Rb2 via C-O. FFEP can be applied to produce minor ginsenosides C-K and C-Y from PPDG-F or TG-F. To the best of our knowledge, this study is the first to report the production of C-K and C-Y from major ginsenosides by basidiomycete *F. fraxinea.*

## 1. Introduction

Ginseng (root of *Panax ginseng* Meyer) has been traditionally applied as an herbal medicine and healthy food for maintenance and restoration of human homeostasis and for enhancement of the immune system, as well as for its adaptogenic and anti-aging properties in East Asian countries, mainly Korea, China and Japan [1,2,3]. Nowadays, it is attracting more attention due to its potential in pharmaceutical and healthy/functional food development. Recent research results reported that ginseng root and processed ginseng products (especially red ginseng extract) have various biological activities, including enhancement of the immune system; anti-fatigue, antioxidant and anti-aging properties; and improvement of blood circulation and climacteric symptoms with anti-tumor, anti-diabetic, neuroprotective and anti-stress effects [4,5,6,7].

Ginseng root contains various biologically active ingredients, including saponins (ginsenosides), polysaccharides and phenolic and nitrogenous compounds [6,8,9]. Among the ginsenosides (dammarane-type and oleanane-type) present in ginseng, dammarane-type ginsenosides can be classified into two types—i.e., protopanaxadiol (PPD)-type and protopanaxatriol (PPT)-type—according to bound position or the number of hydroxyl groups present in their aglycones. Ginsenosides Rb1, Rb2, Rc, Rd, Rh2, C-O, C-Y and C-K belong to the PPD-type ginsenosides group, and Re, Rg1, Rg2 and Rh1 are in the PPT-type group [10,11].

Until recently, more than 80 ginsenosides have been found in ginseng and its processed products [11,12,13]. Among these compounds, ginsenosides Rb1, Rb2, Rc, Re and Rg1 account for about 80–90% of the total ginsenosides, while minor ginsenosides including C-O, C-Y and C-K are present in trace amounts or not detected in raw ginseng [14,15]. However, the absorption of major ginsenosides such as Rb1, Rb2, Rc, Re and Rg1 in the intestinal tract is reported to be difficult due to their large molecular size, poor water solubility and poor cell membrane permeability. In contrast, minor ginsenosides can be effectively absorbed by the human intestine due to their relatively lower molecular size and the ease of cell membrane permeability [16,17,18,19]. Especially, C-K, a major intestinal metabolite of the PPD-type ginsenosides, has been attracting considerable interest due to its various biological activities, including anti-tumor, anti-inflammatory, anti-diabetic, anti-aging, antiallergic, hepatoprotective and anti-skin aging properties [20,21,22,23]. Therefore, many studies have been conducted to convert major ginsenosides into minor ginsenosides by various methods, such as physico-chemical methods [24,25], microbial fermentation and enzymatic biotransformation [26,27,28]. However, physico-chemical methods can bring about the random hydrolysis of attached sugars and unexpected side reactions, including hydration and epimerization [25]. In contrast, enzymatic biotransformation is considered to be a more desirable approach due to high substrate specificity, fewer by-products, *higher biotransformation* efficiency and an eco-friendlier process than physico-chemical methods [26,27].

In particular, ginsenosides Rc and Rb2 have α-L-arabinofuranosyl or α-L-arabinopyranosyl moiety, respectively, at the C-20 terminal position, with three β-glucosyl moieties in PPD-type aglycone. As a result, three types of enzymes (α-L-arabinofuranosidase, α-L-arabinopyranosidase and β-glucosidase) or glycosidases possessing multiple enzyme activity may be required to convert ginsenosides Rb1, Rc or Rb2 into their subsequent minor ginsenosides. However, most of the previously reported studies on the production of minor ginsenosides from major ginsenosides by enzyme-mediated transformation used purified individual ginsenosides or highly purified enzyme sources of high cost. It is therefore preferable to use crude ginseng extracts as a substrate and crude enzyme preparations or whole cells of relatively low cost to economically produce minor ginsenosides from major ginsenosides [26,27,28].

On the other hand, basidiomycetes, commonly known as mushrooms, have received much attention as potentially valuable enzyme sources for production of bioactive compounds [29,30]. Previous studies reported that minor ginsenosides were found to be produced with a high yield from major ginsenosides using crude enzyme preparation obtained from the mycelia of *Armillaria mellea* [31,32]. During our ongoing investigation on biotransformation of major ginsenosides using crude enzyme extracts obtained from various mushrooms, FFEP has a remarkable biotransformation activity of PPD-type major ginsenosides into the minor ginsenosides C-K or C-Y. Basidiomycete *F. fraxinea* (Polyporaceae), which is mainly distributed in the East Asia region, is one of the medicinal mushrooms, and its fruiting body (Korean name; Jangsu mushroom) is traditionally used in Korean folk medicine. In this study, we report on the production of C-K and C-Y from PPDG-F and TG-F containing Rb1, Rc and Rb2 as major ginsenosides by an FFEP-mediated biotransformation method.

## 2. Results and Discussion

### 2.1. β-Glucosidase Activity in FFEP

Mushrooms are not only valuable natural resources for healthy foods and therapeutics, but they are also very interesting fungi that secrete various glycoside hydrolases that can be applicable to biotransformation of various glycosides [29,30,33]. A few studies have reported that fermentation of ginseng extract or ginsenoside-rich fraction by mushrooms could increase the content of minor ginsenosides in fermented products [34,35,36]. These results demonstrate that the enzymes from different strains of mushrooms may convert major ginsenosides to minor ginsenosides by their glycoside hydrolases. Our preliminary study revealed that FFEP has a potent activity for biotransformation of PPD-type ginsenosides (Rb1, Rc and Rb2).

**Figure 1:**
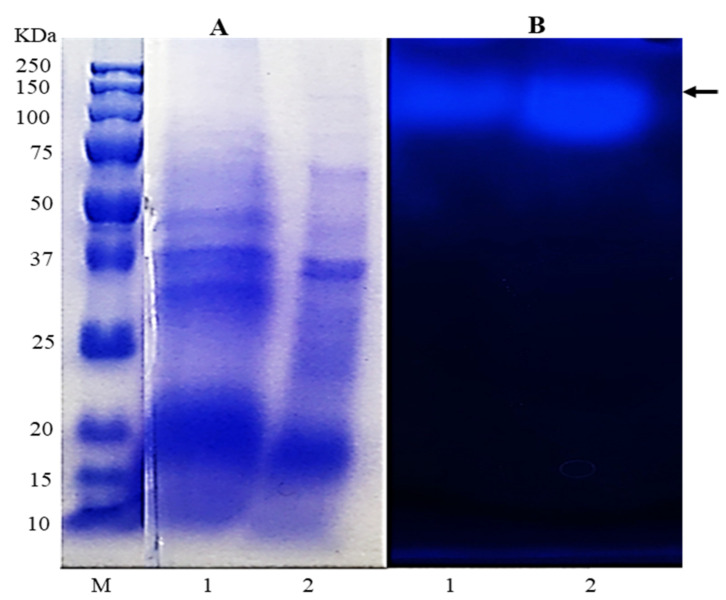
Results of SDS-PAGE (**A**) and native PAGE (**B**) of crude enzyme preparation from *F. fraxinea* grown in submerged culture. M, protein molecular weight marker; 1, total extract; 2, crude enzyme preparation by ammonium sulfate (30–80%) precipitate (FFEP).

As shown in Figure 1, the result of SDS-PAGE and native PAGE showed that FFEP contained β-glucosidase, and its molecular weight was estimated to be between 100 and 150 kDa based on relative migration distance of protein molecular weight markers in electrophoresis. The β-glucosidase activity in FFEP toward *p*-NP-β-D-glucopyranoside was 0.45 U/mg (as a dry weight base). Based on their substrate specificity, microbial β-glucosidases can also be divided into three groups—i.e., group Ⅰ, which possesses only aryl β-glucosidase activities; group Ⅱ, which possesses cellobioses; and group Ⅲ, which contains β-glucosidases showing wide substrate specificity [37]. Among them, the β-glucosidases that belong to group Ⅲ can hydrolyze α-1,3, α-1,4 and α-1,6 glycosidic linkages as well as β-1,2, β-1,4 and β-1,6 glycosidic linkages. The results of the present study show that the β-glucosidase from FFEP used in this study has enzymatic properties belonging to group Ⅲ type β-glucosidase rather than to group Ⅰ or Ⅱ.

### 2.2. Biotransformation of PPDG-F by FFEP

#### 2.2.1. UPLC/ESI-Q-TOF-MS Analysis

**Figure 2:**
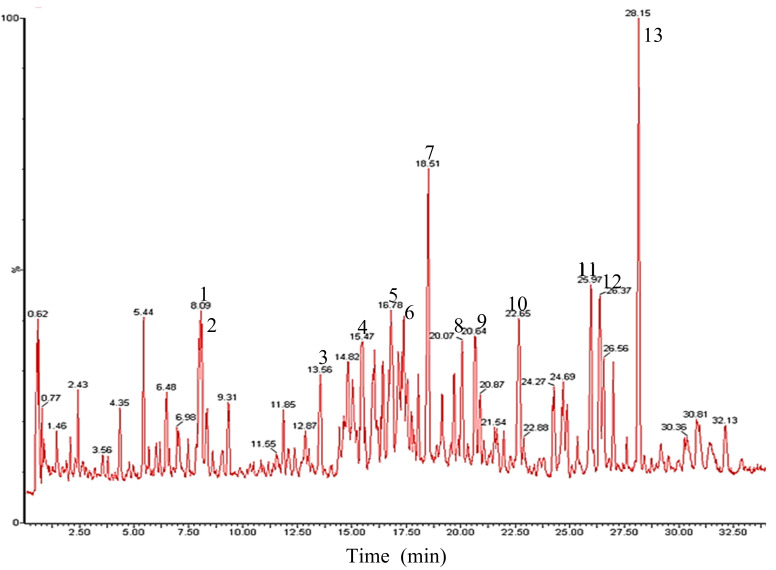
Total ion chromatogram of UPLC/negative-ESI-Q-TOF-MS of biotransformation product of PPDG-F by FFEP.

Recently, UPLC/Q-TOF-MS has been efficiently applied for the structural identification of ginsenosides [38,39]. In the present study, the reaction mixture of PPDG-F with FFEP was withdrawn after 8 h reaction and then was analyzed by UPLC/negative-ESI-Q-TOF-MS to characterize the biotransformation products. The total ion current chromatogram (TIC) is shown in Figure 2.

The identifications of constituents detected in the reaction mixture were also compared with those of individual ginsenoside standards. The results are listed in Table 1. The largest peak 13 in the TIC chromatogram showed protonated ion peaks at *m*/*z* 621.6896 [M − H]^−^ and *m*/*z* 667.7121 [M − H + HCOOH]^−^, corresponding to the molecular formula C_36_H_62_O_8_ with molecular weight (MW) of *m*/*z* 622.4445. From these results, the compound of peak 13 was determined as C-K. Peak 11 and 12 showed the same molecular ion peaks at *m*/*z* 753.7594 [M − H]^−^ and *m*/*z* 753.7576 [M − H]^−^, which corresponded to C_41_H_70_O_12_ as the MW, respectively.

Accordingly, both compounds were estimated as C-Mc and C-Y originating from ginsenosides Rc and Rb2, respectively. Peaks 8 and 9 in Q-TOF–MS analyses were observed as ion peaks at *m*/*z* 915.8387 and *m*/*z* 915.8281 [M − H]^−^ in addition to *m*/*z* 961.8601 and *m*/*z* 961.8512 [M − H + HCOOH]^−^, which corresponds to C_47_H_80_O_17_ as the MW, respectively. In addition, HPLC retention times of peaks 8 and 9 were consistent with those of authentic standards of C-Mc1 and C-O, respectively, Therefore, peak 8 and 9 were estimated as C-Mc1 and C-O, respectively. Similarly, peaks 7, 10, 11, 12 and 13 were elucidated as ginsenoside Rd, F2, C-Mc, C-Y and C-K, respectively, by comparing the Q-TOF-MS data and HPLC retention time with those of reliable standards.

#### 2.2.2. The Effect of Reaction Time

**Figure 3:**
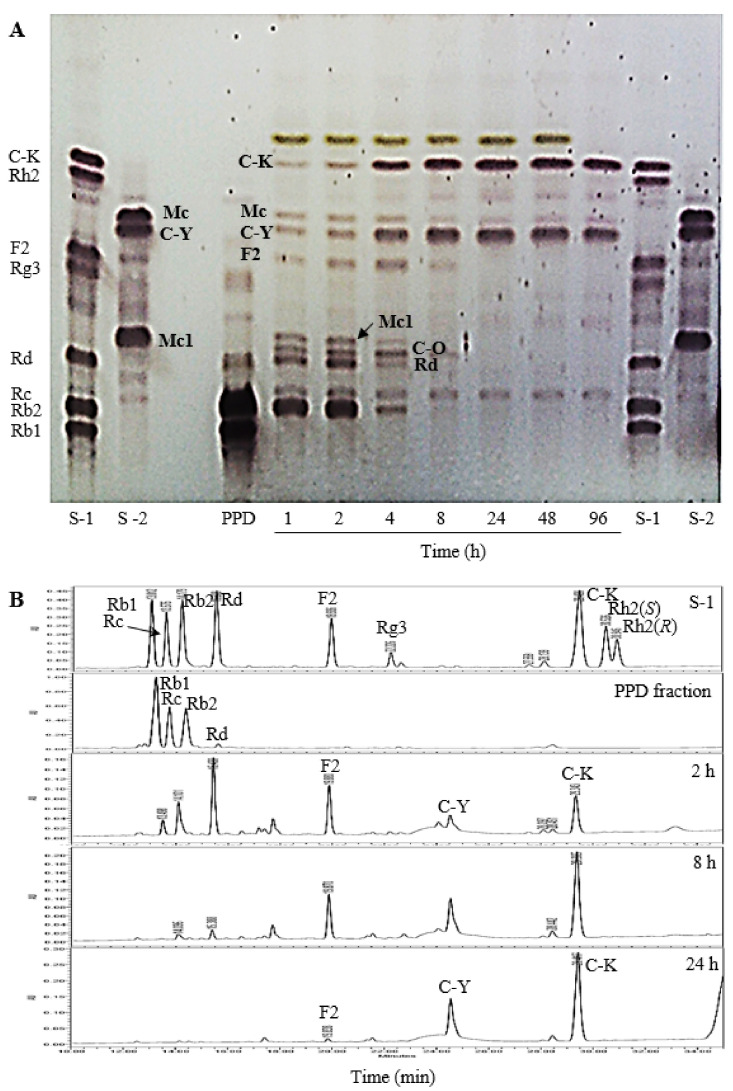
Time course of biotransformation of PPDG-F by FFEP. (**A**) TLC; (**B**) HPLC chromatogram.

During biotransformation of PPDG-F by FFEP, it was regularly monitored by TLC (Figure 3A) and HPLC (Figure 3B). Ginsenosides Rb1, Rc and Rb2 were transformed into their intermediate products, mainly ginsenoside Rd, F2, C-Mc1 or C-O, between 1 and 4 h of reaction, and thereafter, these compounds were transformed into C-K or C-Y by increasing the *reaction time*. However, the concentration of C-K and C-Y increased continuously and reached maxima at 8 h of reaction, and thereafter, *no* meaningful *changes were observed*. These results suggest that FFEP can be used as an enzyme source to produce C-K and C-Y or their mixture from PPDG-F.

#### 2.2.3. The Effect of Reaction pH

**Figure 4:**
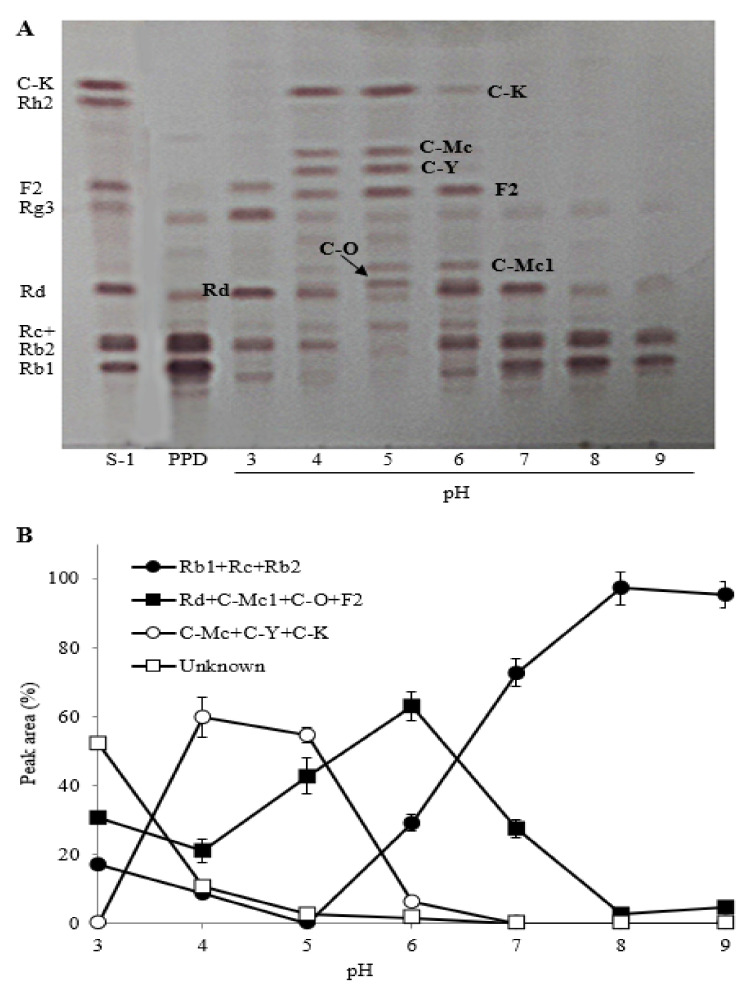
Effect of reaction pH on the biotransformation of PPDG-F by FFEP. (**A**) TLC; (**B**) relative peak area percentage (%) by HPLC.

The biotransformation of PPDG-F by FFEP was investigated at a range from pH 3 to 9. The TLC analysis showed that the optimum biotransformation of Rb1, Rc and Rb1 occurred between pH 4 and 5 (Figure 4). Under this pH range, most of the ginsenoside Rb_1_, Rc and Rb2 were transformed into C-K and C-Y via Rd, F2, C-Mc1 or C-O. PPD-type ginsenosides in acidic buffer solution (pH 3) were hydrolyzed into unknown compounds, which did not match with the R*f* values of authentic standards on TLC (Figure 4A). Ginsenosides can be easily deglycosylated and dehydrated at the C-20 position of aglycones when heated under mild acidic condition [24,25]. When each reaction mixture was investigated by HPLC analysis, most of the parent ginsenosides (Rb1, Rc and Rb2) were biotransformed into minor ginsenosides (C-Mc, C-Y or C-K) between pH 4 and 5, whereas efficient biotransformation could not occur in neutral and alkaline ranges (pH 6–9) (Figure 4B). These results suggest that the biotransformation of PPD-type ginsenosides by FFEP was more desirable at weak acidic condition (pH 4–5) rather than at neutral and basic conditions. Ginsenoside-hydrolyzing enzymes from different microbial sources differ from each other in terms of enzymatic properties and the mode of ginsenoside hydrolysis. Many microbial enzymes showed optimal ginsenoside-hydrolyzing activities at pH 4–5, but neutral β-glycosidases have also been reported from *Pythium irregular*, *Fusobacterium* sp. *Bacteroides* sp. and recombinant β-glucosidase from *Microbacterium esteraromaticum*, with an optimal pH range of 6.5–7.0 [21,26].

### 2.3. Biotransformation of TG-F by FFEP

#### 2.3.1. The Effect of Reaction Time

**Figure 5:**
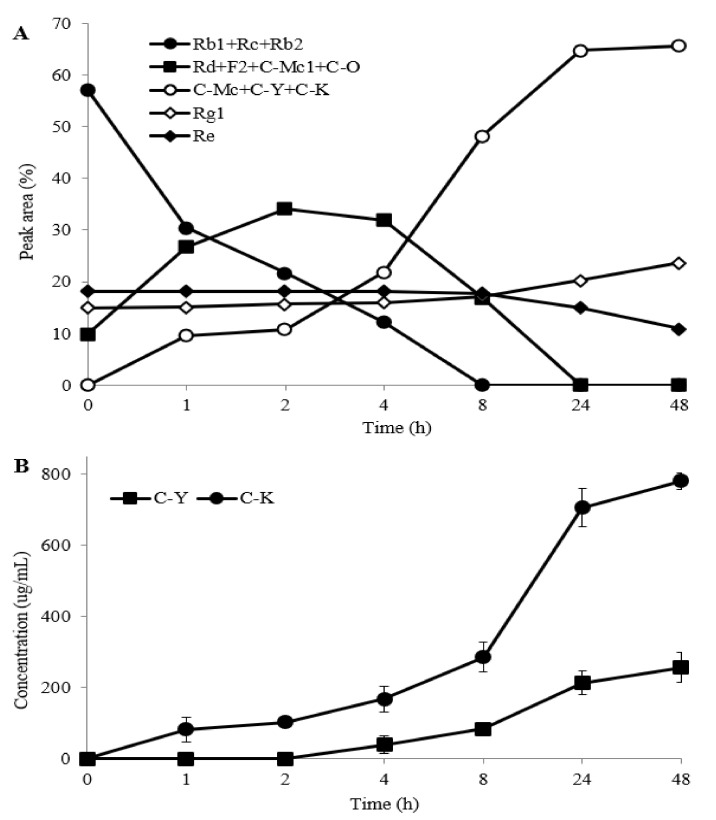
Time course of the biotransformation of total ginsenoside fraction by FFEP. (**A**) relative peak area percentage (%) of each compound by HPLC analysis; (**B**) concentrations of C-K and C-Y.

Reaction mixture samples were taken at different time intervals to investigate the biotransformation properties of TG-F by FFEP and were analyzed by HPLC. As shown in Figure 5A, ginsenosides Rb1, Rc, Rb2 and Rd that comprised the major portion of TG-F were rapidly transformed into F2, C-Mc1 and C-O in the early stage of the reaction (1–4 h). After 8 h reaction, most of the parent ginsenosides (Rb1, Rc, Rb2) and intermediate products (Rd, F2, C-Mc and C-O) were transformed into C-K and C-Y. When the products after 22–48 h reaction were analyzed by HPLC, C-K and C-Y were mainly observed, but C-Mc was hardly detected. These results suggest that C-Mc1 and C-Mc formed from ginsenoside Rc were further hydrolyzed to C-K. Figure 5B shows the time course for the production of C-K and C-Y from TG-F by FFEP. The contents of C-K and C-Y increased with increasing reaction time and yielded 0.780 mg C-K and 0.26 mg C-Y, respectively, from 2 mg TG-F/mL after 48 h reaction.

**Figure 6:**
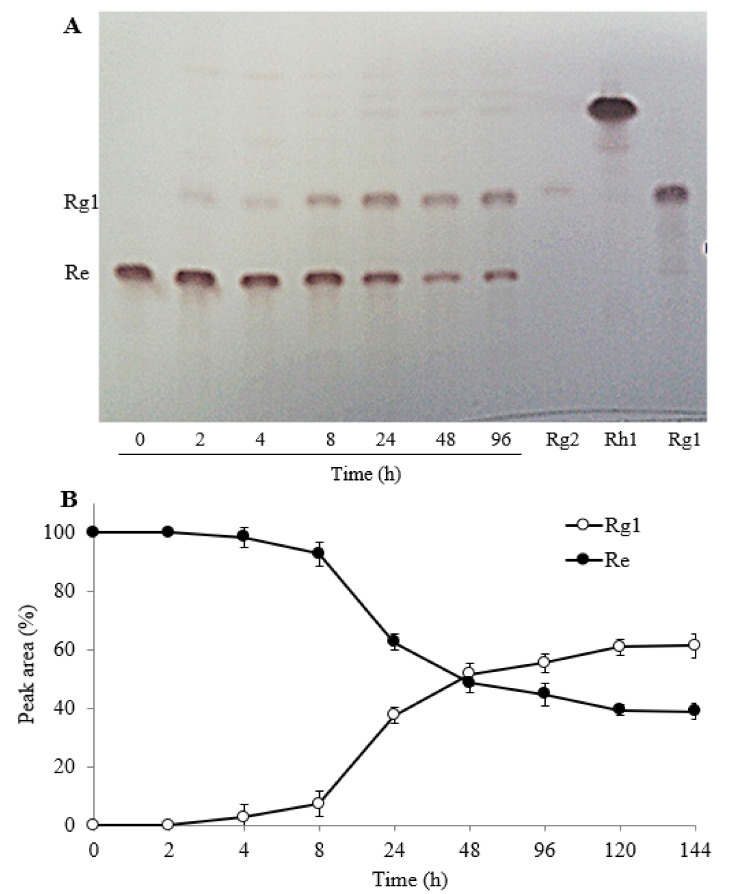
Time course of biotransformation of ginsenoside Re by FFEP. (**A**) TLC; (**B**) relative peak area percentage (%) of ginsenoside Re and Rg1 by HPLC analysis.

On the other hand, during biotransformation of TG-F by FFEP, ginsenoside Re, one of the major PPT-type ginsenosides, was gradually transformed into ginsenoside Rg1 with increasing reaction time. To verify whether PPT-type ginsenosides Re and Rg1 can be hydrolyzed by FFEP, the reaction mixture of Re or Rg1 with FFEP was taken at regular time intervals during 144 h reaction in acetate buffer solution (pH 4.5) and then was analyzed by TLC and HPLC. Ginsenoside Re was gradually transformed into Rg1 in a time-dependent manner (Figure 6A). When ginsenoside Re was used as a substrate (1 mg/mL), about 50% of ginsenoside Re after 48 h reaction was transformed into Rg1 and 60% was transformed after 144 h (Figure 6B). However, additional biotransformation of ginsenoside Rg1 into Rh1 or F1 was not observed.

#### 2.3.2. Biotransformation Pathway of Ginsenosides

**Figure 7:**
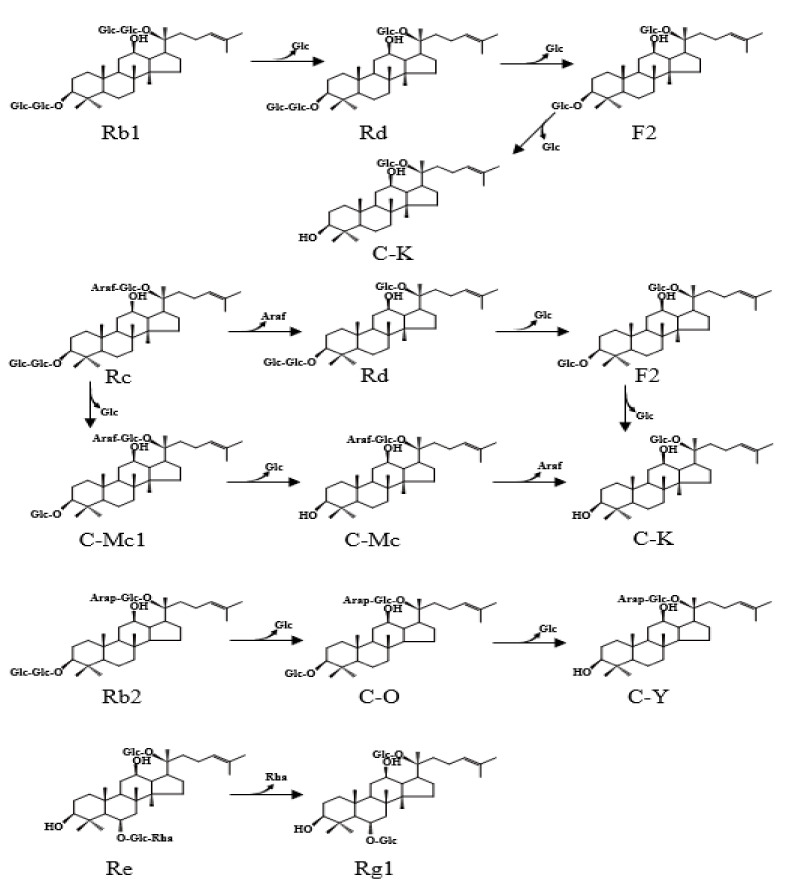
Proposed biotransformation pathways of major ginsenosides by FFEP.

Ginsenoside Rc contains one α-arabinofuranosyl moiety with three β-glucosyl moieties, and Rb2 contains one α-arabinopyranosyl moiety with three β-glucosyl moieties, whereas Rb1 contains four β-glucosyl moieties in PPD-type aglycone, respectively. Therefore, the biotransformation pathways may depend on the stereospecific preferences of the enzymes toward the glycosidic linkages attached to the C-3 and C-20 positions of the aglycone [26]. As shown in Figure 7, the biotransformation of ginsenosides by FFEP is thought to occur along the following pathways: Rb1 → Rd → F2 → C-K for Rb1 as a substrate, both Rc → Rd → F2 → C-K and Rc → C-Mc1 → C-Mc → C-K for Rc and Rb2 → C-O → C-Y for Rb2.

The preference in biotransformation pathways of PPD-type ginsenosides also depends on microbial sources. Biotransformation of Rb1 into C-K can occur through pathways of two types, depending on its structure. One pathway is through hydrolysis of β-(1→6)-glucosyl moiety at the C-20 position and β-(1→2)-glucosyl moiety at the C-3 of aglycone for the formation and Rd and F2 as the intermediates. Another one is by sequentially hydrolyzing the β-(1→2)-glucosyl (outer) and β-glucosyl moieties (inner) at the C-3 position of aglycone for the formation of gypenoside XVII and gypenoside LXXV as the intermediates. Microbial β-glucosidases from *Fusodobacterium* K-10, *Paecilomyces bainier*, *Sulfolobus solfataricusm*, *Cladosporium fulvum* and *Aspergillus oryzae* transformed ginsenoside Rb1 into C-K through the pathway of Rb1 → Rd → F2 → C-K, while *Terrabacter ginsenosidimutans* performed the transformation via the Rb1 → gypenoside ⅩⅦ → gypenoside LⅩⅩⅤ → C-K pathway [21,26]. Ginsenoside Rc can also be hydrolyzed via multiple pathways by microbial β-glucosidases. The results from the present study show that biotransformation of ginsenoside Rc by FFEP occurred through two pathways, as presented in Figure 7. First, FFEP hydrolyzed the α-(1→6)-arabinofuranosidic linkage (outer) linked to the C-20 position of the aglycone to produce Rd from Rc, followed by hydrolysis of β-(1→2)-glucosidic (outer) and β-glucosidic (inner) linkages linked to the C-3 position to produce C-K. At the same time, this enzyme preparation hydrolyzed β-(1→2)-glucosidic (outer) and inner β-glucosidic linkages linked to the C-3 position to produce C-Mc1 and C-Mc from Rc. Thereafter, C-Mc was further hydrolyzed into C-K as a final product.

The ginsenoside Rb2 molecule contains one α-(1→6)-arabinopyranosyl (outer) and one β-glucopyranosyl moiety (inner) at the C-20 position, with two β-glucopyranosyl moieties at the C-3 position of aglycone. Accordingly, Rb2 can also be hydrolyzed by multiple pathways. Based on the results obtained by TLC and HPLC analysis, we suggest that Rb2 by FFEP was biotransformed into C-Y via C-O (Figure 7). This enzyme attacked the outer β-(1→2)-glucosidic linkage linked to the C-3 position of aglycone to produce C-O from Rb2 and then followed by hydrolysis of the inner β-glucosidic linkage linked to the C-3 position to produce C-Y from C-O. In this study, ginsenoside Re, one of the PPT-type ginsenosides, was gradually converted into ginsenoside Rg1 (Figure 7). This result indicates that FFEP possessed hydrolytic activity toward β-glucosyl (1-1)-α-rhamnosyl linkage at the C-6 position of the PPT-type aglycone. Until now, enzymatic transformation of PPT-type ginsenosides were rarely reported [28,40,41]. However, FFEP had no hydrolytic activity toward β-glucosidic linkages at the C-6 or C-20 positions of ginsenoside Rg1.

## 3. Materials and Methods

### 3.1. Reagents

β-D-Glucopyranoside, α-L-arabinofuranoside, α-L-arabinopyranoside of *p*-nitrophenol (*p*-NP) as chromogenic substrates, bovine serum albumin (BSA), sodium dodecyl sulfate (SDS), 4-methylumbelliferyl-β-D-glucopyranoside (MUG) and dialysis membrane tubing (14,000 cutoff) were purchased from Sigma-Aldrich Korea (Gangnam-gu, Seoul, Korea). Mini protean TGX precast gel and protein molecular markers and Coomassie blue R-250 were purchased from Bio-Rad Laboratories, Inc. (Kangnam-gu, Seoul, Korea). Silica gel TLC plate was purchased from Merck KGaA (Darmstadt, Germany). Deionized water was manufactured by using a water purification system (model New Human Power I, Human Corp., Songpa-ku, Seoul, Korea). Acetonitrile and methanol (HPLC grade) were purchased from Avantor Performance Materials Korea (Suwon, Gyeonggi-do, Korea). Other reagents were obtained from a commercial source (Daejung Chemical Co., Siheung, Gyonggi-do, Korea).

### 3.2. Preparation of Ginsenosides and Ginseng Extracts

Authentic standards (ginsenosides Rb1, Rb2, Rc, Rd, Rg3, F2, Rh2, C-K, Re and Rg1), PPDG-F and TG-F were kindly provided by the Korea Ginseng Research Institute (Daejeon, Korea). The compositions of Rb1, Rc, Rb2 and Rd in the PPDG-F were 50.8, 21.2, 27.7 and 0.3%, respectively, as peak area in HPLC analysis. Authentic standards of C-Mc1, C-Mc, C-O and C-Y were prepared from ginsenoside Rc or Rb2 using crude enzyme preparation isolated from the mycelial mass of *Armillaria mellea* according to our previously reported methods [31,32]. Separately, ginsenoside Rb1, Rc and Rb2 were also isolated from PPDG-F according to a previously reported method [10]. The isolated compounds were identified by comparison of NMR and Q-TOF-MS data and HPLC retention times with those of authentic standards.

### 3.3. Microorganism

Strain of *F. fraxinea* (KACC 42289) was provided by the Korean Agricultural Culture Collection (KACC) of Rural Development Administration (Wanju, Jeonbuk, Korea). The strain was kept on an agar slant media including 2% malt extract at 4 °C.

### 3.4. Mushroom Cultivation

Strain of *F. fraxinea* was previously incubated on potato dextrose agar for 2 weeks at 24–25 °C. After the incubated strain was inoculated into saccharified malt media (11°Brix), it was cultivated for 2 weeks at 24–25 °C with orbital shaking (120 rpm). Scale-up cultivation of the strain was performed in a 3-L Erlenmeyer flask containing 1 L of malt media (11°Brix) for 2 weeks at 24–25 °C with shaking (120 rpm). All nutrient media were sterilized at 121 °C for 30 min.

### 3.5. Preparation of Crude Enzyme

The cultivated liquid culture with mycelial mass was homogenized at maximum speed with a homogenizer (Omni International, Kennesaw, GA, USA) for 1 min and then centrifuged at 12,000× *g* for 20 min. After the supernatant was treated with ammonium sulfate powder (30% saturation) for 6 h, the solution was centrifuged to remove the protein precipitate and then ammonium sulfate was added in the supernatant to 80% saturation and maintained overnight to induce the protein precipitate including β-glucosidase and followed by centrifuging (12,000× *g*) for 20 min. After the precipitate was dissolved in a small volume of 10 mM sodium acetate buffer (pH 4.5) and purified by overnight dialysis with 10 mM acetate buffer (pH 4.5), the supernatant obtained was lyophilized for 4 days. All experiments were carried out at 4 °C unless otherwise mentioned.

### 3.6. Enzyme Electrophoresis

SDS-polyacrylamide gel electrophoresis (PAGE) was done with a precast gel (12%) for 1 h at 110 mA according to Laemmli’s method [42]. The gel was stained with Coomassie blue R-250 and destaining of the stained gel was performed with distilled water containing methanol and acetic acid (each 10%, *v*/*v*). Native PAGE was performed with a precast gel (12%) under the same conditions as above. Thereafter, the gel was maintained in acetate buffer (0.1 M, pH 4.5) containing 0.1% MUG (*w*/*v*) for 30 min at 37 °C. The band due to aglycone (*4*-*methylumbelliferol*) *liberated in the gel was detected under UV light* (365 nm).

### 3.7. Enzyme Activity and Protein Assays

β-Glucosidase activity was assayed according to the method of Mfombep et al. [30], with slight modifications. The reaction mixture (1.0 mL)—containing 0.1 mL of *p*-NP-β-D-glucopyranoside (10 mM) as a substrate, enzyme solution (0.1 mL) and 0.1 M acetate buffer (pH 4.5) (0.8 mL)—was incubated for 30 min at 37 °C. After termination of the reaction by adding 0.5 M sodium carbonate (1.0 mL), the liberated *p*-nitrophenol (aglycone) was measured using a UV-Vis spectrophotometer (UV-1601, Shimadzu, Kyoto, Japan) at 400 nm. The amount of *p*-nitrophenol liberated was quantified based on a calibration curve prepared using an authentic standard. An amount of 1 unit of enzyme activity was defined as the amount of enzyme required to release 1 μM *p*-nitrophenol per min. Protein content was assayed according to the Bradford method, using BSA as a standard [43].

### 3.8. Enzymatic Biotransformation

The reaction mixtures (2.0 mL) each containing 2.0 mg of PPDG-F or TG-F in methanol (0.2 mL) and FFEP possessing 2.7 U as β-glucosidase activity (6.0 mg, 0.45 U/mg) in 1.8 mL of 0.1 M acetate buffer (pH 4.5) were incubated at 45 °C with shaking (90 rpm), respectively. The reaction mixtures were sampled periodically and then heated for 10 min in boiling water to inactivate the enzyme, followed by extraction twice (each 2 mL) with saturated *n*-butanol with water. After the butanol extract was washed with water (twice) to minimize residual sugars, the solution was subjected to dryness in *vacuo*. The residue was dissolved in 1.0 mL methanol, and was analyzed by TLC, HPLC or ultra-performance liquid chromatography/negative-time-of-flight mass spectrometry (UPLC/Q-TOF-MS). The effect of pH on the biotransformation of PPDG-F by FFEP was incubated for 24 h at 45 °C in the following buffers (each at 0.1 M): glycine-HCl (pH 3), sodium acetate (pH 4 and 5), sodium phosphate (pH 6 and 7), Tris-HCl (pH 8), and glycine-NaOH (pH 9). Separately, the mixture (20 mL) containing TG-F (20 mg) in methanol (2 mL) and FFEP containing β-glucosidase (27 U, 60 mg) in 18 mL of 0.1 M acetate buffer (pH 4.5) was reacted for 48 h at 45 °C. The reaction mixture (2.0 mL) was sampled periodically and then digoxin (480 μg/mL) was added as an internal standard, followed by extraction twice with saturated *n*-butanol with water. The residues after vacuum evaporation were dissolved in methanol (1.0 mL).

### 3.9. General Analytical Methods

The analyses by TLC, HPLC and UPLC/negative-ESI-Q-TOF-MS were conducted as described in our previous studies [31,32]. The ratio of each peak in HPLC analysis was calculated based on relative percentage of the detected peak area. The concentrations of C-K and C-Y produced during biotransformation of TG-F were quantified by the internal standard method using digoxin as an internal standard without considering the detector response factor. HPLC analyses for quantitation of C-K and C-Y in FFEP-mediated reaction mixtures of TG-F were done in triplicate, and the results are presented as average ± standard deviation (SD).

## 4. Conclusions

Production of minor ginsenosides by using highly purified major ginsenosides or purified enzymes is an economically disadvantaged, *complicated* and *time*-*consuming process*. Crude enzyme preparation isolated from submerged culture of *F. fraxinea* containing β-glucosidase exhibited potent biotransformation activity toward PPD-type ginsenosides such as Rb1, Rc and Rb2. Furthermore, ginsenoside Re was converted into Rg1, resulting in a transformation yield of about 50% after 48 h reaction. The results of the present study suggest that FFEP can be used to produce valuable minor ginsenosides C-K and C-Y from PPDG-F or TG-F, with high biotransformation efficiency. However, *further studies are necessary* to optimize the cultivation condition of this mushroom strain, improve the *recovery* of the *enzyme* from a *liquid culture*, as well as optimize the ratio of enzyme-to-substrate for improving the biotransformation yield.

## Figures and Tables

**Table 1:** UPLC/Q-TOF-MS analysis of biotransformation products from PPDG-F by FFEP.

Peak no.	RT (min)	Identification	[M − H]^−^		[M − H + HCOOH]^−^	Molecular Formula
			Theoretical Mass	Detected Mass		
1	8.09	G-Rg1	799.4845	799.7836	845.7795	C_42_H_72_O_14_
2	8.11	G-Re	945.5248	945.8526	991.8694	C_48_H_82_O_18_
3	13.56	G-Rf	799.4815	799.7791	845.7988	C_42_H_72_O_14_
4	15.47	G-Rh1	637.4316	637.4324	683.1782	C_36_H_62_O_9_
5	16.78	G-Rc	1077.5781	1077.9037	1123.9282	C_53_H_90_O_22_
6	17.31	G-Rb2	1077.5683	1077.9022	1123.9231	C_53_H_90_O_22_
7	18.51	G-Rd	945.5248	945.8588	991.8710	C_48_H_82_O_18_
8	20.07	C-Mc1	915.5396	915.8387	961.8601	C_47_H_80_O_17_
9	20.64	C-O	915.7124	915.8281	961.8512	C_47_H_80_O_17_
10	22.65	G-F2	783.4895	783.7744	829.7908	C_42_H_72_O_13_
11	25.97	C-Mc	753.4844	753.7594	799.7759	C_41_H_70_O_12_
12	26.37	C-Y	753.4789	753.7576	799.7819	C_41_H_70_O_12_
13	28.15	C-K	621.4366	621.6896	667.7121	C_36_H_62_O_8_

## Data Availability

The data that support the findings of this study are available from the corresponding author, upon reasonable request.

## Sample Availability

Samples of the compounds are available from the authors.
